# Resilience Factors Preventing Schizophrenia in Ultra-high Risk Patients: Lessons from Genetics

**DOI:** 10.1192/j.eurpsy.2022.49

**Published:** 2022-09-01

**Authors:** B. Chaumette, C. Jiao, Q. He

**Affiliations:** University of Paris, INSERM, U1266 Institute of Psychiatry and Neuroscience of Paris, Paris, France; GHU Paris Psychiatrie et Neurosciences, PEPIT, U1266 Institute of Psychiatry and Neuroscience of Paris, Paris, France; McGill University, Department of Psychiatry, Montréal, Canada

## Abstract

Over the past decades, researchers and psychiatrists in the field of psychosis have moved from a conception of a chronic presentation to a more dynamic paradigm. Accordingly, schizophrenia is now conceptualized as a progressive illness that typically emerges during late adolescence and follows different stages: early vulnerability, ultra-high risk state, first episode of psychosis, and chronic disease. Only one-quarter of the ultra-high risk patients will convert to a full-blown psychotic episode within 3 years while the others, called non-converters, will remain at-risk, develop other psychiatric disorders, or fully recover. The reasons for this differential outcome are not yet understood but this concept opens the way to scientific research to determine the protective factors involved in resilience for non-converters. Based on the Gene X Environment interaction model, schizophrenia results from genetic vulnerability and environmental aggressions which can have an impact on the epigenome and gene expression. Recent studies have shown that genetic variants play a role in the resilience of psychosis. Polygenic risk scores, computed as the addition of genetic polymorphisms, can modulate the effects of genetic at-risk deletions (i.e. del22q11) that predispose to psychosis and may also influence the cognitive symptoms of ultra-high risk patients. Resilience, defined as the ability to withstand adversity, is not only related to external skills or psychotherapeutic care but could also be explained by internal molecular factors. Identifying the genetic factors of resilience might help to stratify the risk and to develop precision medicine in psychiatry.

**Disclosure:**

No significant relationships.

